# Bioinformatics and systems biology research update from the 15^th^ International Conference on Bioinformatics (InCoB2016)

**DOI:** 10.1186/s12859-016-1409-7

**Published:** 2016-12-22

**Authors:** Christian Schönbach, Chandra Verma, Peter J. Bond, Shoba Ranganathan

**Affiliations:** 1 International Research Center for Medical Sciences, Graduate School of Medical Sciences, Kumamoto University, Kumamoto, 860-0811 Japan; 2 Bioinformatics Institute, Agency for Science, Technology and Research (A∗STAR), Singapore, 138671 Singapore; 3 Department of Chemistry and Biomolecular Sciences, Macquarie University, Sydney, NSW 2109 Australia

**Keywords:** InCoB, International Conference on Bioinformatics, APBioNet, Asia-Pacific Bioinformatics Network

## Abstract

The International Conference on Bioinformatics (InCoB) has been publishing peer-reviewed conference papers in BMC Bioinformatics since 2006. Of the 44 articles accepted for publication in supplement issues of BMC Bioinformatics, BMC Genomics, BMC Medical Genomics and BMC Systems Biology, 24 articles with a bioinformatics or systems biology focus are reviewed in this editorial. InCoB2017 is scheduled to be held in Shenzen, China, September 20–22, 2017.

## Introduction

InCoB2016, the 15^th^ International Conference on Bioinformatics was held in Singapore from September 21^st^ to 23^rd^, 2016 [1]. An update on the annual conference of Asia-Pacific Bioinformatics Network [2] including review process and best paper awards is available in the introduction to the InCoB2016 supplement issue of BMC Genomics [3]

The papers introduced in this introduction are arranged by topics: (1) sequence analysis and ontologies; (2) networks and systems biology; (3) protein structural bioinformatics, (4) ligand design and ligand-target interactions, and (5) bioimaging.

## Sequence analysis and ontologies

The presentations in this session covered a variety of topics ranging from algorithm and development to motif finding. Abbas and Bahig [4] investigated the partial digestion problem by proposing two algorithm "branch and bound based on breadth" (BBb) and "branch and bound based on breadth two times" (BBb2). The latter requires less memory than BBb as the partial digestion problem is solved in two stages in breadth-first mode. Gan et al. [5] have developed a comparative transcriptomic analysis webserver, PARRoT, for non-reference organisms, using a homologue-based virtual transcriptome reference, which provided homologues from sequence databases as well as gene ontology (GO) terms. For entire microbial communities, Xie et al. [6] have developed RiboTagger, for fast and accurate recovery of small subunit ribosomal RNA sequences, from all three domains of life. Zheng et al. [7] profiled small RNAs in soybean primary root tips under conditions of water deficiency. The study reported 22 novel miRNAs and one miRNA which was significantly down-regulated under water deficiency.

Molecular recognition factors (MoRFs) located within longer intrinsically disordered proteins perform important cellular functions such as signalling and regulation, but are very difficult to accurately detect. Sharma et al. [8] have addressed this challenge using Hidden Markov Model (HMM) profiles, with their results better with other similar approaches, MoRFpred and ANCHOR. Vasylenko et al. [9] have developed SCMBYK, to predict and characterise bacterial tyrosine kinases from protein sequence information, using dipeptide propensity scores. Their analysis of bacterial tyrosine kinases from *Mycobacetrium tuberculosis* could explain how azathioprine could suppress the pathogenicity of this organism in transplant patients. Le and Ou [10] have developed an excellent model for predicting guanosine triphosphate (GTP) binding sites in transport proteins using radial basis function networks, trained on position specific scoring matrix profiles and significant amino acid pairs. This predictive model is available to the community as a webserver.

Gutierrez and Nakai [11] have built a correlated topic model to find transcription factor binding motifs, which enhanced the performance of their earlier model significantly. Lan et al. [12] analysed the grouping miRNAs of similar functions using a weighted gene ontology information content. The authors demonstrated improved clustering performance of miRNA into subgroups of similar functions and utility in annotating new miRNAs.

## Networks and systems biology

Castiglione et al. [13] have developed a minimalist gene regulatory network model to simulate macrophage differentiation into the pro-inflammatory and the anti-inflammatory phenotypes. The model was tested in a statistical ensemble and showed robust gene regulatory logic as well as overall dynamics. The authors plan to extend this model to simulate gene knockouts and ectopic expressions, to better understand macrophage biology. To comprehend the regulatory behaviour of microRNAs (miRNAs), Lee and Lee [14] propose a new framework for identifying direct miRNA-mRNA association networks, called DMirNet, which can contribute to the construction of direct regulatory pathways, by identifying novel miRNA-mRNA interactions. In order to characterize cell-specific gene expression as well as gene co-expression. At the single cell level, Ghazanfar et al. [15] have developed a versatile modelling framework for transcriptional state analysis and coactivation detection across multiple datasets. Their results on olfactory neurons enable unique cell-specific coactivation network delineation. At the biological system level, Pennisi et al. [16] have used Petri nets to model the immune system. In particular, coloured Petri nets have provided features for modelling signalling pathways, improving on the classical discrete and continuous models.

## Protein structural bioinformatics

One of the fundamental assumptions of structural bioinformatics is that hydrophobic residues prefer to be buried in protein structures. By analysing the standard Barton502 dataset using support vector regression on informative physicochemical properties, Liou et al. [17] show that an aligned row (called spline) of solvent-exposed hydrophobic residues actually stabilise ɑ-helices in proteins. Su et al. [18] have used structural bioinformatics approaches to correlate recent clinically identified mutation in HIV-1 protease with different protease inhibitors used as drugs. Their results provide an insight into the mechanism for HIV-1 drug resistance and suggest a protease inhibitor drug may selection strategy for clinical applications.

## Ligand design and drug-target interactions

Small organic molecules as drug lead compounds is an important area of structural bioinformatics research, where knowledge of the target’s 3D structure enables rational ligand design, for therapeutic applications. Using a series of derivatives from the influenza drug zanamivir, Dholakia et al. [19] have generated group-wise quantitative structure activity relationships (QSAR) for inhibiting the neuraminidase protein from H1N1 and H3N2 influenza strains. Their model was then applied to identify a new lead compound with drug-like properties and binding stably to both H1N1 and H3N2 neuraminidase active sites. GQSAR with combinatorial design has been adopted by Joshi et al. [20] to identify two novel inhibitors for casein kinase 1 δ isoform, as potential drugs for neuronal protection in amyotrophic lateral sclerosis.

Given the spiralling cost of drug development, a growing number of approved drug molecules are being considered for medical conditions different from their original purpose, known as repurposing or repositioning. As the number of known drug-target interactions far exceeds the potential interactions of these drugs with other potential targets, predicting the repurposing of a drug appears to be extremely challenging. Ezzat et al. [21] have addressed this class imbalance problem using an ensemble learning approach to successfully predict several new drug-target interactions. Sun et al. [22] have addressed the same challenge using a Physarum-inspired Prize-Collecting Steiner Tree algorithm, to build drug similarity networks, from which ten frequently occurring drug molecules have been reported as potential new cardiovascular therapeutic agents.

## Bioimaging

Computer-based morphological analysis present a rapid and efficient way to analyse biological samples. Skull analysis can be used for clinical applications as well as for species classification. Based on human orthodontic parameters used in clinical analysis, Mosleh et al. [23] have developed Ceph-X, for automated cephalometric analysis, with excellent concordance with expert manual analysis results. On the other hand, Abu et al. [24] have developed a novel, rapid and accurate computer-assisted taxonomical classification system for the house shrew, with seven morphological parameters and artificial neural networks (ANN). Kalafi et al. [25] have extended cephalometric analysis to species classification, using images of hard parts of the haptoral organs of flatworms, such as bars and anchors and K-nearest neighbour approach. Such an approach is important for monitoring species density and mobility, for biodiversity informatics and ecological modelling.

In pathology applications, cell-based microscopic analysis suffer from out-of-focus images due to depth effects. Intarapanich et al. [26] have developed an object-based extended depths of field (OEDoF) processing approach, which is more accurate and much faster than the state-of-the-art complex wavelet algorithm. Their method has been applied to malaria samples and suitable for medical diagnostic applications. Multiple reporter gene tagging provides fluorescence in different colours for tracking biological processes. However, segmentation issues in identifying cell boundaries in multicolour genetic labelling strategies pose serious problems. Nguyen et al. [27] have developed a novel strategy to overcome these issues, by integrating an edge detector into a superpixel framework with customization for multi-channel images. Their method has been applied successfully to muscle fibres and can be adapted for stem cell regeneration and cell lineage tracking.

## Conclusion

The quality of the articles reviewed in this introduction are comparable with earlier InCoB conferences while the subject coverage has extended substantially into bioimaging. Next year's conference, InCoB2017 will be held in Shenzhen from Sept. 20–22, 2017 [28], with paper submissions on Easychair available from January 2017.

## Abbreviations

APBioNet: Asia-Pacific Bioinformatics Network
BBb: Branch and bound based on breadth
BBb2: Branch and bound based on breadth two times
GO: Gene ontology
GTP: Guanosine triphosphate
HIV-1: Human immunodeficiency virus 1
InCoB: International Conference on Bioinformatics
OEDoF: Object-based extended depths of field
QSAR: Quantitative structure activity relationships
